# Health literacy as a mediator of the relationship between socioeconomic status and health: A cross-sectional study in a population-based sample in Florence

**DOI:** 10.1371/journal.pone.0227007

**Published:** 2019-12-23

**Authors:** Vieri Lastrucci, Chiara Lorini, Saverio Caini, Florence Health Literacy Research Group, Guglielmo Bonaccorsi

**Affiliations:** 1 Department of Health Science, University of Florence, Florence, Italy; 2 Cancer Risk Factors and Lifestyle Epidemiology Unit, Institute for Cancer Research, Prevention and Clinical Network (ISPRO), Florence, Italy; Catholic University of Korea College of Medicine, REPUBLIC OF KOREA

## Abstract

**Background:**

Health literacy(HL) has recently been proposed as a potential mediator in the pathway through which socio-economic status(SES) affects health. However, empirical research investigating the contribution of HL in this relationship remains scarce. This study investigated whether functional HL mediates the association between SES and self-reported health(SRH) in an adult population-based sample.

**Methods:**

The study adopted a cross-sectional design. Education level and financial status were used as measures of SES, while functional HL was assessed with the Newest Vital Sign. Moderated mediation analyses were conducted using SES variables as independent variables, SRH as dependent variable and functional HL as mediator variable. Furthermore, age, sex and chronic diseases were tested as moderators of the effect mediated by functional HL.

**Results:**

452 subjects completed the study (58,8% female; mean age 53,25±11,7). Results showed that functional HL mediates on average 18.5% of the association between education and SRH (*p* = 0.02) and 12.9% (*p* = 0.01) of the association between financial status and SRH. Furthermore, the proportion of effect mediated by functional HL was found to be higher in lower socio-economic classes for both SES variables considered. No significant moderation effects of age, sex or chronic diseases were observed for both SES variables.

**Conclusion:**

Findings suggest that functional HL may serve as a pathway by which SES affects health status, especially in lower SES groups. HL may be a valuable and actionable intermediate target for addressing health inequalities. However, further studies are needed to better define the mediating role of HL across socio-economic classes.

## Introduction

The scientific literature has repeatedly demonstrated that socioeconomic factors are powerful determinants of health-related outcomes, and socioeconomic status (SES) may be considered as one of the main causes of health disparities between different population groups [1–3]. However, SES does not directly affect health status, rather it represents a proxy of other proximal and intermediate causal factors. Several competing factors and mechanisms have been proposed to explain the chain of events linking SES to health outcomes [1]. However, the entire pathway by which SES exerts its effect on health has not yet been completely elucidated [4].

Recently, health literacy (HL) has been proposed as one of the potential links between SES and health [5–8]. According to Sørensen and colleagues, HL is linked to literacy and can be defined as the “people’s knowledge, motivation and competences to access, understand, appraise and apply health information in order to make judgments and take decisions in everyday life concerning healthcare, disease prevention and health promotion to maintain or improve quality of life during the life course” [9]. Therefore, HL appears to be in close correlation with both socio-economic determinants and health outcomes. In a recent study that assessed both antecedents and consequences of HL in the same sample, Bonaccorsi et al [10] found that less educated and poorer population groups have more often limited or inadequate HL and that low HL skills are associated in turn with worse self-reported health status. Indeed, on the one hand, disadvantaged socio-economic groups have been reported to have a greater risk of limited HL compared with more advantaged socio-economic groups [11–13]. On the other hand, a limited HL has been shown to be associated with unhealthy lifestyle behaviors and several negative health-related outcomes, such as higher mortality and hospitalization rates, greater use of emergency care, lower receipt of mammography screening and influenza vaccine and poorer management of chronic diseases [14–18]. However, most studies published to date have separately analyzed predictors and outcomes of HL, while only a few have evaluated the possible contribution of HL in the pathway through which socio-economic determinants affect health [19].

Thus far, only one systematic review on the role of HL in the relationship between SES and health has been published [19]. Results of this review suggest that HL partially mediates the effect that SES exerts on health status and on several health related-outcomes. However, the review pointed out that the evidence on the mediating role of HL is scarce, and that data from population-based sample studies are lacking. Yet, given the difficulties to act directly on socio-economic determinants of health, the confirmation of the mediating role of HL may help design and implement health policies and interventions aimed at reducing health inequalities.

While assessing the mediation effect, it is also important to determine if results are equal across population subgroups. Indeed, the effect of SES determinants on health via HL might vary depending on the values taken by one or more third variables. Specifically, these third variables–defined as effect moderators [20]—may affect (either enhance or reduce) the relationship between SES and HL and/or the relationship between HL and health. Therefore, the mediating role of HL in the pathway through which SES determinants affect health may be strengthened or weakened depending on the values taken by the moderator. From a public health perspective, the identification of the moderators may help target the population subgroups who would benefit most from HL interventions. However, to the best of our knowledge, no studies have analyzed the moderators of the mediating role of HL in the relationship between socio-economic factors and health.

The aim of the present study is to evaluate whether functional health literacy mediates the association between socio-economic factors and health status in a population based-sample.

## Materials and methods

The study was approved by the Ethics Committee of the “*Area Vasta Centro*” (Local Health Unit of Central Tuscany, Careggi University hospital and Meyer University Children’s Hospital; Ref. CEAVC:10113, 01 December 2016) and was conducted according to the principles described in the Declaration of Helsinki. This study is part of a research project conducted to assess the level of HL in a population-based sample in Florence, and to validate different HL measures in Italian language. For more detailed information regarding methods and projects outputs, the reader is referred to the study protocol and articles published elsewhere [10, 21–22].

### Study population and sampling criteria

This is a cross-sectional study carried out in a not-representative population-based sample. Participants were randomly selected from a list of residents available from the registers of eleven general practitioners (GPs) working in primary healthcare centers of the municipality of Florence, Italy. According to the regulations of the National Healthcare System and the Constitution of the Italian Republic, every Italian and foreign resident aged ≥18 years has to be registered in a general practice, and people are enrolled in the general practices according to their place of residence (percentage of resident population registered 98.8%). This sampling method was chosen with the aim of increasing the participation rate as the invitation letter was jointly signed by the general practitioners and the researcher in charge of the study [23]. The GPs were recruited using convenience criteria. All the GPs of the municipality of Florence were invited to join the study by both the Provincial Medical Council and the University Hospital of Florence. A total of 11 GPs based in different districts of Florence were recruited on a first-come basis. The number of GPs recruited in the study was increased from what had been originally proposed in the study protocol (i.e. n = 8) in order to extend the geographical coverage of the study [21]. The recruited general practitioners were based in the city center and in the inner and outer suburban areas of Florence.

Each GP selected 80 people from his/her register through a random number generator. Inclusion criteria were the following: 18–69 years of age (this criterion was adopted in order to be coherent with the Italian lifestyle surveillance system [24]) and Italian speaking (since the survey was conducted in Italian only). Exclusion criteria included severe cognitive impairment and severe psychiatric diseases as these conditions may significantly impair the ability to understand the questions and provide reliable answers. Furthermore, in line with the principles described in the Declaration of Helsinki, people with end-stage diseases were excluded as all the potential insights derived from the research would not be beneficial to this population group. Inclusion and exclusion criteria were applied by each GP independently.

### Data collection, main variables and HL measure

Data were collected between February and December 2017. Each selected person was contacted via postal mail. The selected people received a mail that included a short description of the study, an invitation to participate and a consent form. Participants were asked to sign the consent form and return it via mail to the researchers in charge. The mail also contained the nutritional label of the Newest Vital Sign-Italian (NVS-IT) designed to be easily readable (i.e. large font size and line-spacing). After receipt of the signed consent form, each participant was contacted by phone for the computer-assisted interview. If the consent form was not received within two weeks, a follow-up phone call was made by the research group. The phone call served to clarify any questions and to identify and assist people with difficulties in completing the consent form (e.g. reading difficulties). Nine interviewers conducted the phone interviews. A shared written protocol on how to conduct the interview was followed in order to standardize the interviews and to limit interviewer bias. Each participant was randomly assigned to one of the nine interviewers and contacted up to six times before being considered unreachable. The whole interview took about 20–25 minutes.

Several demographic, socioeconomic, and health outcome variables were collected. For the specific purpose of the present study the following variables were utilized: sex, age, presence of chronic diseases, education level, financial status, and SRH. In particular, age in years was calculated from birth year; the presence of chronic diseases—defined as any disease that has lasted or is expected to last for at least 6 months—were coded in five categories: yes, more than one; yes, one; no; do not know; refuse to answer. Each participant’s education level was defined as the highest level of education attainment and was categorized as follows: Bachelor’s degree or higher; high school diploma; lower secondary school diploma or lower. As for the financial status, it was assessed by the item “is your income adequate to meet monthly living expenses?”, response options being: more than adequate; adequate; barely adequate; inadequate; refusal. Finally, SRH was chosen as it represents a valid and valuable measure of the overall health status [25–28] that is widely employed in general population health surveys [25]. SRH was assessed by a single item: “In general, would you say that your health is excellent, very good, good, fair, or poor?”.

Functional health literacy was assessed using the NVS-IT tool. The NVS-IT consists of an ice cream nutrition label, with seven associated questions that measure literacy and numeracy. It produces a final score ranging from 0 to 6, allowing participants to be classified into three levels of functional HL: high likelihood of limited HL (score: 0–1), possibility of limited HL (score: 2–3), and adequate HL (score: 4–6).

### Statistical analysis

Education level, financial status and SRH were analyzed as dichotomous variables. Specifically, education level was re-classified into the following categories: *i*. Bachelor degree or higher and *ii*. high school diploma or lower. The following two categories for financial status were defined: *i* adequate or more than adequate income and *ii*. barely adequate / inadequate income to meet monthly living expenses. SRH was classified into the following two categories: *i*. good SRH (*i*.*e*. excellent, very good, and good SRH) and *ii*. fair or poor SRH. Lastly, for all the analyses, subjects with high likelihood of limited HL and those with possibility of limited HL were grouped together in a single group, referred to “inadequate and at-risk HL” and compared with those with adequate HL [15].

**Fig 1:**
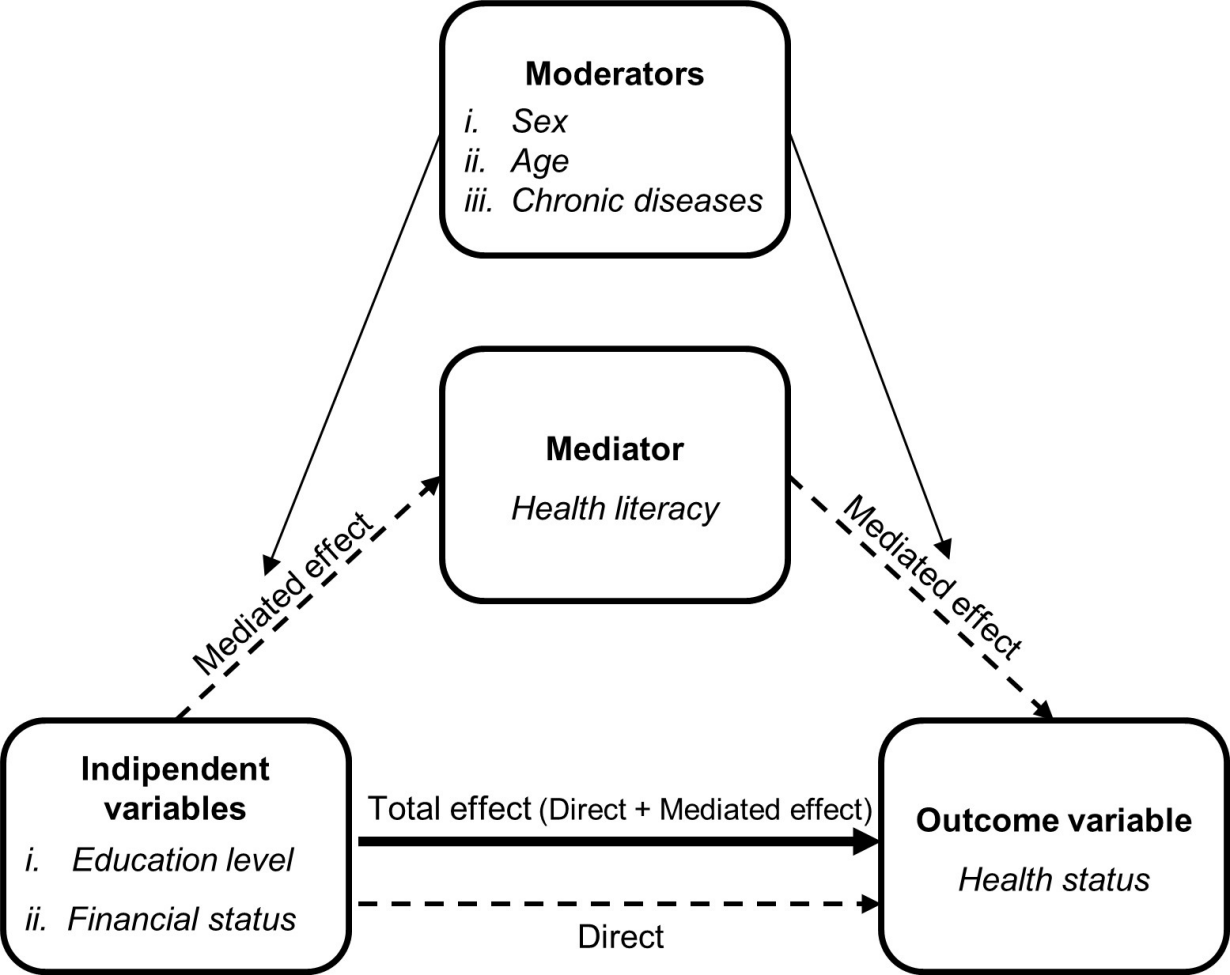
Conceptual model of health literacy as a mediator of the association between socio-economic factors and health status.

In the mediation analysis, a pathway is specified a priori in which an independent variable of interest influences an outcome through an intermediate variable, which is referred to as a mediator (see Fig 1). Specifically, to test whether HL mediates the association between socio-economic factors (*i*.*e*. education level and financial status) and health status, a model-based causal moderated mediation analysis was performed separately for each of the two socioeconomic factors considered [29].

The analysis proceeded in two steps. First, two logistic regression models were specified, *i*. *the mediator model* for the conditional distribution of the mediator (health literacy levels) given the independent variable (socio-economic factor), and *ii*. *the outcome model* for the conditional distribution of the outcome (self-reported health status) given the independent variable and the mediator. These models were fitted separately and controlled for age and sex as covariates; in addition, the outcome model also contained an interaction term for the independent variable x the mediator. Subsequently, the outputs of the mediator and outcome regression models served as the main inputs to the “mediate function” (mediation package in R software version 4.4.6) [29], which computes the total effect of the independent variable on the outcome and decomposes this effect into the indirect (*average causal mediation effects*—*ACME*) and direct (*average direct effects*- *ADE*) effects. Specifically, the indirect effect reflects one possible explanation of the observed effect (*i*.*e*. that transmitted through the mediator), whereas the direct effect represents all other possible causal chains. Furthermore, the mediate function estimates the proportion of the effect mediated, which describes the average magnitude of indirect association between the independent variable and the outcome that is due to changes in the mediator variable relative to the average total association. Lastly, “moderated mediation” models were fitted in order to identify whether the magnitude of the ACME of HL varies depending on (*i*.*e*. “is moderated by”) the values taken by other covariates (defined as “moderators”). The following variables were tested as potential moderators: age, sex, and the presence of one or more chronic diseases. In the analyses, age was used as a continuous variable, and multiple comparisons between age classes were made. As for chronic diseases, the following comparisons were tested: no *vs*. one chronic disease; no *vs*. two or more chronic diseases; and one *vs*. two or more chronic diseases.

As the missing values were lower than 1% for all the variables considered in the moderated mediation models, a complete data analysis with no attempt to input missing values was performed. For each analysis, an α level below 0.05 was considered as significant.

## Results

### Descriptive statistics of the sample

A total of 984 individuals were invited to participate in the study, of which 493 agreed to be interviewed (50.1%) and 454 (46.1%) were effectively interviewed. As far as non-participation reasons were concerned, 340 (34.5% of the total sample) people did not respond to any contact attempts, 151 (15.3% of the total sample) people refused to participate, and a further 39 (4% of the total sample) people initially agreed to be interviewed, but subsequently it was not possible to arrange an interview. Non-participants were on average two year younger than participants (51.2 ± 11.8 years and 53.3 ± 11.7 years, respectively). No significant sex differences emerged between participants and non-participants. Two interviewees were excluded from the study because of missing data on several variables; therefore, a total sample of 452 participants were finally included in the analyses.

Table 1 shows selected participants’ characteristics. Females represented 58.8% of the sample and participants with bachelor’s degree or higher represented the 41.1% of the sample; 69.9% of the participants had an income adequate or more than adequate to meet monthly living expenses. As for health status, 31% of the participants referred a very good or excellent health status. In terms of HL, high likelihood of limited HL, possibility of limited HL and adequate HL were found in 11.5%, 24.6% and 63.9% of the sample, respectively.

**Table 1:** Characteristics of the sample.

	Total n(% for column)	Health Literacy (HL) levels n(% for raw)		
		High likelihood of limited HL	Possibility of limited HL	Adequate HL
All	452(100)	52(11.5)	111(24.6)	289(63.9)
Female	266(58.8)	30(11.3)	62(23.3)	174(65.4)
Male	186(41.2)	22(11.8)	49(26.3)	115(61.8)
Age	53.25±11.72	59.44 ± 9.61	57.79±9.88	50.40±11.77
**Chronic diseases**				
No	237(52.4)	27(11.4)	50(21.1)	160(67.5)
One	147(32.5)	11(7.5)	43(29.2)	93(63.3)
More than one	67(14.8)	14(20.9)	17(25.4)	36(53.7)
Refusal	1(0.2)	0(0.0)	1(100)	0(0.0)
**Education level**				
Bachelor’s degree or higher	186(41.1)	9(4.8)	33(17.7)	144(77.4)
High school diploma	192(42.5)	26(13.5)	49(25.5)	117(60.9)
Less than high school diploma	74(16.4)	17(23.0)	29(39.2)	28(37.8)
**Financial status**				
More than adequate/adequate	316(69.9)	29(12.8)	75(16.7)	212(70.5)
Barely adequate/inadequate	132(29.2)	23(17.4)	34(25.8)	75(56.2)
Refusal	4(0.9)	0(0.0)	2(50.0)	2(50.0)
**Self-reported health status**				
Excellent	27(6)	3(11.1)	4(14.8)	20(74.1)
Very good	113(25)	9(8)	17(15)	87(77)
Good	208(46)	21(10.1)	53(25.5)	134(64.4)
Fair	93(20.6)	15(16.1)	32(34.4)	46(49.5)
Poor	10(2.2)	4(40)	4(40)	2(20)
Refusal	1(0.2)	0(0)	1(100)	0(0)

### Association between health literacy and independent variables (mediator models)

The results of the mediator models for education and financial status are shown in Tables 2 and 3, respectively. Both education level and financial status were positively associated with HL. In particular, participants with a high school diploma or lower education level had an increased odds ratio (OR) of having inadequate or at-risk HL compared with those with bachelor’s degree or higher education level (OR 2.59, 95%C.I. 1.66–4.02). As for the relationship between financial status and HL, participants with inadequate or barely adequate income had an increased odds ratio of having inadequate or at-risk HL compared with those with adequate or more than adequate income to meet the monthly living expenses (OR 2.03, 95%C.I. 1.28–3.21).

**Table 2:** Mediator model, outcome model and moderated mediation analysis for education level.

Mediator model* Dependent variable: *“inadequate or at-risk” vs*. *“adequate” HL*	**Odds Ratio**	**95% C.I.**	***p***
Bachelor’s degree or higher	1	-	*-*
High school diploma or lower	2.59	1.66–4.02	*<0*.*01*
Outcome model* Dependent variable: *“fair or poor” vs*. *“good” SRH*	**Odds Ratio**	**95% C.I.**	***p***
*Health Literacy (HL)*			
Adequate HL	1	-	*-*
Inadequate or at-risk HL	1.90	1.17–3.10	*0*.*01*
*Education level*			
Bachelor’s degree or higher	1	-	*-*
High school diploma or lower	1.76	1.06–2.91	*0*.*03*
Moderated mediation analysis*	***B***	**95% C.I.**	***p***
Total Effect	0.109	0.029–0.185	*0*.*01*
Average causal mediation effect (ACME)	0.021	0.004–0.043	*0*.*01*
Average direct effect (ADE)	0.088	0.008–0.164	*0*.*04*
Proportion mediated (average)	18.5%	3.1% - 71.7%	*0*.*02*
Proportion Mediated (high school diploma or lower)	21.1%	3.8% - 73.6%	*0*.*02*
Proportion Mediated (bachelor’s degree or higher)	15.8%	2.5% - 72.1%	*0*.*02*

*models adjusted by age and sex

**Table 3:** Mediator model, outcome model and moderated mediation analysis for financial status.

Mediator model* Dependent variable: *“inadequate or at-risk” vs*. *“adequate” HL*	**Odds Ratio**	**95% C.I.**	***p***
Adequate/more than adequate financial status	1	-	*-*
Inadequate/barely adequate financial status	2.03	1.28–3.21	*<0*.*01*
Outcome model* Dependent variable: *“fair or poor” vs*. *“good” SRH*	**Odds Ratio**	**95% C.I.**	***p***
*Health Literacy (HL)*			
Adequate HL	1	-	*-*
Inadequate or at-risk HL	3.75	1.68–8.37	*<0*.*01*
*Financial status*			
Adequate or more than adequate	1	-	*-*
Inadequate or barely adequate	4.08	2.00–8.34	*<0*.*01*
*Interaction term*	0.32	0.12–0.86	*0*.*02*
Moderated mediation analysis*	***B***	**95% C.I.**	***p***
Total Effect	0.164	0.079–0.254	*<0*.*01*
Average causal mediation effect (ACME)	0.022	0.003–0.047	*0*.*01*
Average direct effect (ADE)	0.143	0.062–0.226	*<0*.*01*
Proportion mediated (average)	12.9%	2.4% - 31.3%	*0*.*01*
Proportion mediated (barely adequate or inadequate)	24.0%	3.7% - 58.1%	*0*.*02*
Proportion mediated (adequate or more than adequate)	2.0%	-6.0% - 16.0%	*0*.*54*

*models adjusted by age and sex

### Association between health status, health literacy and independent variables (outcome models)

The results of outcome models for education and financial status are shown in Tables 2 and 3, respectively. As for the former, lower HL and educational level were both independently associated with an increased odds of reporting a worse health status (OR 1.90, 95%C.I. 1.17–3.10; and OR 1.76, 95%C.I. 1.06–2.91, respectively). As for outcome model for financial status, participants with inadequate or barely adequate income and participants with inadequate or at-risk HL levels were found to have an increased odds ratio of worse health status (OR 4.08, 95%C.I. 2–8.34; and OR 3.75, 95%C.I. 1.68–8.37, respectively).

No significant interaction between HL and education level was seen in the former model, and the interaction term was therefore not retained in the model whose output was entered in the moderated mediation analysis. Instead, there was a negative interaction between HL and financial status in the latter case (OR for the interaction term 0.032, 95%CI 0.12–0.86).

### Mediation effect of health literacy and analyses of the moderators

In Table 2 is presented the mediation effect of health literacy in the association between education level and SRH. On average, HL was found to significantly mediate 18.5% of the association between education and SRH; in particular, HL significantly mediated 21.1% of the association in participants with high school diploma or lower education levels and 15.8% of the association in participants with bachelor’s degree or higher education level. As far as the relationship between financial status and SRH is concerned (Table 3), HL was found to significantly mediate 12.9% of the association on average and 24% of the association in participants with inadequate or barely adequate income; the mediating role of HL was not significant in participants with adequate or more than adequate income (*p* = 0.54).

As for the analyses of moderators, the mediating role of HL in the association between education and SRH was not significantly moderated by age, sex and chronic diseases (p> 0.05, results not reported). Similar results were observed in the analysis of moderators of the association between financial status and SRH: the mediation effect of HL was not significantly moderated by age, gender and chronic diseases (p> 0.05, results not reported).

## Discussion

The aim of the study was to evaluate whether functional health literacy constitutes a pathway by which socio-economic determinants affect health. This hypothesis was tested in a population-based sample using education level and financial status as socio-economic determinants, self-reported health as outcome measure, health literacy as mediator, and age, sex or presence of chronic diseases as potential effect moderators. Results of logistic regression models showed the existence of positive associations among socio-economic determinants, functional HL and SRH. In particular, lower education and worse financial status were independently associated with a worse SRH and with lower functional HL levels, and inadequate or at-risk HL emerged as a predictor of poor SRH. The mediation analysis models showed that functional HL partly mediates the effect of the socio-economic determinants on health, and that the proportion of the overall effect of socio-economic determinants on SRH mediated by functional HL is higher among study participants belonging to lower socio-economic classes. Lastly, no moderation effects of age, sex or presence of chronic diseases on the proportion mediated by functional HL were found, suggesting that the observed pattern is robust across demographic characteristics and “objective” health status.

As regards the interrelationships among socio-economic determinants, HL and health, results of our study are in line with the scientific evidences available to date. Indeed, as for the relationship between SES and health, our findings confirm that SES disparities predict health disparities [3,30–33]; also in accordance with the literature, our findings confirm that SES and HL are positively linked and that HL is a predictor of health status [11–13,17,34]. These findings confirm the need of exploring the potential mediating role of HL in the relationship between socio-economic factors and health investigated by the present study and by few other studies [19].

Regarding the mediating role of HL in the relationship between socioeconomic factors and health status, our findings showed that functional HL may constitute one of the possible pathway by which socio-economic factors influence health status. This result is in line with the literature, although it should be pointed out that only very limited studies have investigated the mediating role of HL to date [19]. Should this result be confirmed in futures studies, HL would be entitled to be listed together with the other established mediators that link SES determinants to health status such as behaviors and lifestyles, social and environmental exposures and access to, use of, and quality of health care [1, 35–36]. Besides deepening the research concerning the underlying mechanisms linking SES to health status, the mediating role of HL may have important implications for the interventions aimed at reducing health disparities as HL can be modified more easily than the SES determinants. Policies and interventions aimed at increasing the level of HL in the population or that take people’s low HL into account might effectively contribute to reduce health inequalities.

Furthermore, the results of the moderated mediation analysis highlighted that functional HL constitutes a more important pathway among those with a lower socio-economic status than among those with higher socio-economic status, in which functional HL resulted to play a limited (and possibly, not significant) role. A similar finding was reported by the study of Van der Heide and collaborators in which the mediation effect of health literacy resulted to play a larger role in lower education levels [37]; however, due to the paucity of data to date, further research is needed to confirm the presence of a socio-economic gradient in the mediating role of HL. The analysis of the relative weight of the mediating role of HL among socio-economic classes may help identify population groups that may benefit most from intervention aimed at improving HL.

As far as the analysis of moderators is concerned, the proportion of mediated effect by functional HL was not found to vary according to the participants’ age, sex, or presence of chronic diseases, suggesting that the role of functional HL as a mediator of the effect of socio-economic determinants on health status is relatively constant across population subgroups defined in terms of demographics and objectively measured health status. However, it should be underlined that only few effect moderators have been explored in this study and that this study is the first to examine effect moderators of the mediating role of HL in the literature, to the best of our knowledge. Several other potential moderators may affect the mediating role of HL in the pathway through which SES determinants affect health as several third variables—such as ethnicity, occupation, perceived social status or specific chronic diseases [8,15,38–40]—may potentially influence the relationship between SES and HL and/or the relationship between HL and health. As the identification of moderators of the mediating role of HL may help to identify population subgroups who will benefit most from targeted HL interventions, further and more in-depth studies on effect moderators are needed to confirm our results and to explore the presence of other effect moderators.

Our study has some limitations that should be acknowledged. First, data cannot be considered representative of the overall Italian or Florentine adult population as the population-based sample was obtained through a combination of convenience and probability sampling procedures; indeed, while participants were randomly selected from a list of residents available from the registers of the GPs, the GPs were selected on a convenience basis. This may represent a major limitation for external comparison of the study results, since the convenience sampling of GPs may have introduced a selection bias. However, since the included GPs were based in different districts of the city, the geographical coverage of the sample included residents of different areas of the city, thus partially counterbalancing the selection bias detailed above. Secondly, as the variables have been self-reported by the participants, a social desirability bias may have affected the accuracy or completeness of the information retrieved, especially for the reported financial status [41]. However, the telephone interview may have limited this potential bias. Thirdly, with regard to self-reported financial status, it should be underlined that the perceived financial status may be influenced by a broad set of factors; in particular, age and economic conditions experienced at the time of labor market entry by each birth cohort may shape the perception of the financial status [42,43]. However, it should be pointed out that the subjective perception of financial status is not only consistently and robustly related to objective financial capacity but also predicts health-related outcomes and SRH even after controlling for objective economic factors [44–47]. Lastly, the study had a high non-participation rate that may be attributable to the participants’ recruitment method—*i*.*e*. postal mail and phone call invitations—as most of the selected people resulted unreachable (no answer). This issue may have introduced a non-participation bias; however, it should be underlined that participants and non-participants differed only moderately in terms of age distribution.

Concerning potential areas for future research, it should be pointed out that only a uni-dimensional aspect of HL (*i*.*e*. functional HL) was assessed in the present study and that the mediating role of HL may be different if assessed with the use of multidimensional HL measures [48]. Indeed, different HL dimensions may be differently linked to both socio-economic determinants and health status. The use of multidimensional HL measures would allow a better definition of the role of HL—and its sub-dimensions—as a potential mediator of the effect of socio-economic determinants on health. Another potential area of interest for future research is the evaluation of specific and objective health outcome measures in order to better define the mediating role of HL in the different health domains. Lastly, our study could be further improved by simultaneously taking into account the other potential mediators of the relationship by which socioeconomic factors influence health status in order to evaluate the role and positioning of HL as a mediator in a broader framework [1,35–36].

## Conclusion

Our findings suggest that functional health literacy may serve as a pathway by which socioeconomic status affects health status. If confirmed, these findings would not only provide insight into the underlying mechanisms by which socio-economic disparities contribute to health differences, but would also suggest that functional HL may be a valuable target to address health inequalities. Indeed, HL may be more easily modified compared to the main established socio-economic determinants of health inequalities, on which it is often difficult to act. Policies and interventions aimed at increasing the level of HL in the population (or that otherwise take into account people’s HL) may be an effective means to reduce health inequalities. Therefore, a thorough characterization of the attributes of HL as a mediator of the relationship between socioeconomic status and health is critical in order to orient, tailor and maximize any public health effort aimed at reducing health inequalities. However, there is very limited research on the topic and further studies that take into account possible effect moderators, multidimensional HL measures and specific health outcome measures are needed to better define the role of HL in mediating the relationship between socio-economic determinants and health status.

## Supporting information

S1 Dataset Full dataset of the study. (SAV)
